# Spiking Cortical Model Based Multimodal Medical Image Fusion by Combining Entropy Information with Weber Local Descriptor

**DOI:** 10.3390/s16091503

**Published:** 2016-09-15

**Authors:** Xuming Zhang, Jinxia Ren, Zhiwen Huang, Fei Zhu

**Affiliations:** Department of Biomedical Engineering, School of Life Science and Technology, Huazhong University of Science and Technology, No. 1037, Luoyu Road, Wuhan 430074, China; M201471648@hust.edu.cn (J.R.); M201571646@hust.edu.cn (Z.H.); zhufei@hust.edu.cn (F.Z.)

**Keywords:** multimodal medical image fusion, spiking cortical model, weighting fusion, entropy, Weber local descriptor

## Abstract

Multimodal medical image fusion (MIF) plays an important role in clinical diagnosis and therapy. Existing MIF methods tend to introduce artifacts, lead to loss of image details or produce low-contrast fused images. To address these problems, a novel spiking cortical model (SCM) based MIF method has been proposed in this paper. The proposed method can generate high-quality fused images using the weighting fusion strategy based on the firing times of the SCM. In the weighting fusion scheme, the weight is determined by combining the entropy information of pulse outputs of the SCM with the Weber local descriptor operating on the firing mapping images produced from the pulse outputs. The extensive experiments on multimodal medical images show that compared with the numerous state-of-the-art MIF methods, the proposed method can preserve image details very well and avoid the introduction of artifacts effectively, and thus it significantly improves the quality of fused images in terms of human vision and objective evaluation criteria such as mutual information, edge preservation index, structural similarity based metric, fusion quality index, fusion similarity metric and standard deviation.

## 1. Introduction

With the development of medical imaging technology, various imaging modals such as ultrasound (US) imaging, computed tomography (CT), magnetic resonance imaging (MRI), positron emission tomography (PET) and single-photon emission computed tomography (SPECT) are finding a range of applications in diagnosis and assessment of medical conditions that affect brain, breast, lungs, soft tissues, bones and so on [1]. Owing to the difference in imaging mechanism and the high complexity of human histology, medical images of different modals provide a variety of complementary information about the human body. For example, CT is well-suited for imaging dense structures like non-metallic implants and bones with relatively less distortion. Likewise, MRI can visualize the pathological soft tissues better whereas PET can measure the amount of metabolic activity at a site in the body. Multimodal medical image fusion (MIF) aims to integrate complementary information from multimodal images into a single new image to improve the understanding of the clinical information in a new space. Thus, MIF plays an important role in diagnosis and treatment of diseases and has found wide clinical applications, such as US-MRI for prostate biopsy [1], PET-CT in lung cancer [2], MRI-PET in brain disease [3] and SPECT-CT in breast cancer [4].

Numerous image fusion algorithms have been proposed by working at pixel level, feature level or decision level. Among these methods, the pixel-level fusion scheme has been investigated most widely due to its advantage of containing the original measured quantities, easy implementation and computational efficiency [5]. Existing pixel-level image fusion methods generally include substitution methods, multi-resolution fusion methods and neural network based methods. The substitution methods such as intensity hue saturation [6,7], principal component analysis [8] based methods can be implemented with high efficiency but at the expense of reduced contrast and distortion of the spectral characteristics. Image fusion methods based on the multi-resolution decomposition techniques can preserve important image features better than substitution methods via the decomposition of images at a different scale to several components using pyramid (e.g., contrast pyramid [9] and gradient pyramid [10]), empirical mode decomposition [11] or various transforms including wavelet transform [12,13,14], curvelet transform [15], ripplet transform [16], contourlet transform [17], non-subsampled contourlet transform (NSCT) [18,19,20,21,22] and shift-invariant shearlet transform [23,24]. However, the transform based fusion methods involve much higher computational complexity than the substitution methods, and it is challenging to adaptively determine the involved parameters in these methods for the different medical images.

The various neural networks such as self-generating neural network [25] and pulse coupled neural network (PCNN) have been used for image fusion. Different from some traditional neural networks, PCNN, as the third generation artificial neural network, has biological background and it is derived from the phenomena of synchronous pulse bursts in the visual cortex of mammals [26,27]. The PCNN based MIF method has gained much attention due to its great advantages of being very generic and requiring no training. The parallel image fusion method using multiple PCNNs has been proposed by Li et al. [28]. The multi-channel PCNN (m-PCNN) based fusion method has been proposed by Wang et al. [29], and it has been further improved by Zhao et al. [30]. Recently, PCNN has been combined with multi-resolution decomposition methods such as the wavelet transform [31], the NSCT [32,33,34,35,36], the shearlet transform [37,38] and the empirical mode decomposition [39]. These methods involve such disadvantages as high computational complexity, difficulty in adaptively determining PCNN parameters for various source images and image contrast reduction or loss of image details. In view of high computational complexity of PCNN, Zhan et al. [40] have recently proposed a computationally more efficient spiking cortical model (SCM), a single-layer, local-connected and two-dimensional neural network. Wang et al. [41] have presented a fusion method based on the firing times of the SCM (SCM-F). Despite the superiority of SCM over PCNN in computational efficiency, the SCM-F method will lead to loss of image details during fusion because it only utilizes the firing times of individual neurons in the SCM to establish the fusion rule, and employs a too simple fusion strategy. 

To address the problem of unwanted image degradation during fusion for the above-mentioned fusion methods, we have proposed a distinctive SCM based weighting fusion method. In the proposed method, the weight is computed based on the multi-features of pulse outputs produced by SCM neurons in a neighborhood rather than the individual neurons. The multi-features include the entropy information of pulse outputs, which can characterize the gray-level information of source images, and the Weber local descriptor (WLD) feature [42] of firing mapping images produced from pulse outputs, which can represent the local structural information of source images. Compared with the PCNN based fusion method, the proposed SCM based method using the multi-features of pulse outputs (SCM-M) has such advantages as higher computational efficiency, simpler parameter tuning as well as less contrast reduction and loss of image details. Meanwhile, the proposed SCM-M method can preserve the details of source images better than the SCM-F method. Extensive experiments on CT and MR images demonstrate the superiority of the proposed method over numerous state-of-the-art fusion methods.

The remainder of the paper is structured as follows. Section 2 describes the spiking cortical model. Section 3 presents the details of the proposed SCM-M method. The experimental results and discussions are presented in Section 4. Conclusions and future research directions are given in Section 5. 

## 2. Spiking Cortical Model

**Figure 1:**
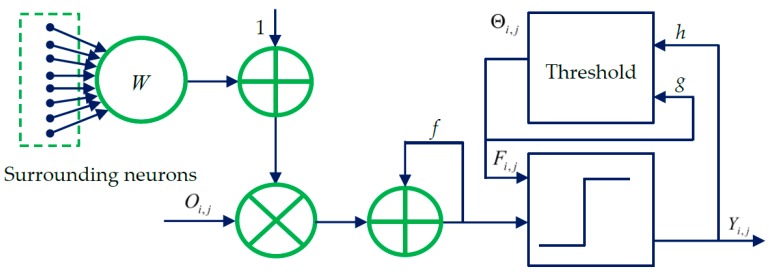
The structural model illustrating the spiking cortical model (SCM).

The spiking cortical model (SCM) is derived from several other visual cortex models such as Eckhorn’s model [26,27]. The SCM has been specially designed for image processing applications. The structural model of the SCM is presented in Figure 1. As shown in Figure 1, each neuron $N_{i,j}$ at (*i*,*j*) corresponds to one pixel in an input image, receiving its normalized intensity as feeding input $O_{i,j}$ and the local stimuli from its neighboring neurons as the linking input. The feeding input and the liking input are combined together as the internal activity $F_{i,j}$ of $N_{i,j}$. The neuron $N_{i,j}$ will fire and a pulse output $Y_{i,j}$ will be generated if $F_{i,j}$ exceeds a dynamic threshold $\Theta_{i,j}$. The above process can be expressed by [40]:

(1) $$F_{i,j}[n]=f\begin{array}{c} F_{i,j}[n-1] \end{array}+O_{i,j}+O_{i,j}\sum_{k,l}W_{i,j,k,l}Y_{k,l}[n-1]$$

(2) $$Y_{i,j}[n]=\left\{ \begin{array}{ll} 1 & if\;F_{i,j}[n]>\Theta_{i,j}[n-1]\\ 0 & otherwise \end{array}\right.$$

(3) $$\Theta_{i,j}[n]=g\begin{array}{c} \Theta_{i,\begin{array}{c} j \end{array}}[n-1] \end{array}+h\begin{array}{c} Y_{i,j}[n-1] \end{array}$$

where *f* and *g* are decay constants less than 1; h is the scalar of large value; *n* denotes the number of iterations ($1\leq n\leq N_{\max}$, $N_{\max}$ is the maximum iteration times); and $W_{i,j,k,l}$ is the synaptic weight between $N_{i,j}$ and its linking neuron $N_{k,l}$ and it is defined as:

(4) $$W_{i,j,k,l}=\left\{ \begin{array}{cc} \frac{1}{\sqrt{{\left(i-k\right)}^{2}+{\left(j-l\right)}^{2}}} & if\;\left(i,j)\neq (k,l\right)\\ 0 & otherwise \end{array}\right.$$

**Figure 2:**
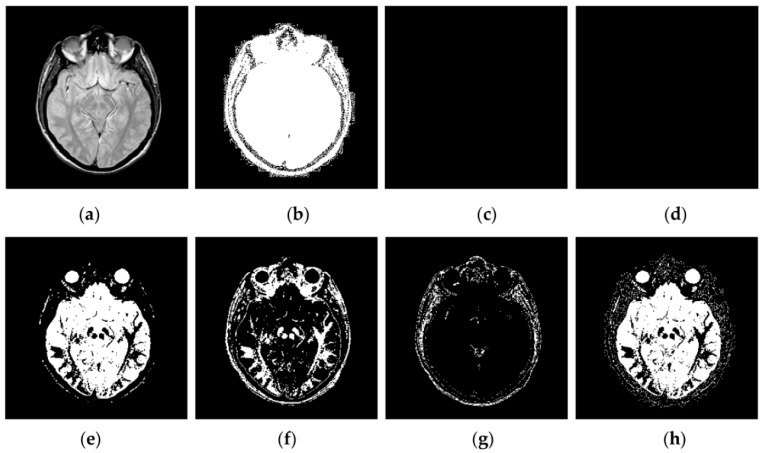
Temporal series of pulse outputs generated by the SCM operating on magnetic resonance (MR) image: (**a**) MR image; and (**b**–**h**) the binary pulse images from the first to the seventh iteration, respectively.

Through iterative computation, the SCM neurons output the temporal series of binary pulse images. The temporal series contain much useful information of input images. To explain this point better, Figure 2 shows the temporal series produced by the SCM with *f* = 0.9, *g* = 0.3, *h* = 20 and *N_max_* = 7 operating on an input MR image shown in Figure 2a. In Figure 2, we can see that during the various iterations, the output binary images contain different image information and the outputs of the SCM typically represent such important information as the segments and edges of the input image. The observation from Figure 2 indicates that the SCM can describe human visual perception. Therefore, the pulse outputs of the SCM can be utilized for image fusion.

## 3. SCM Based Image Fusion

**Figure 3:**
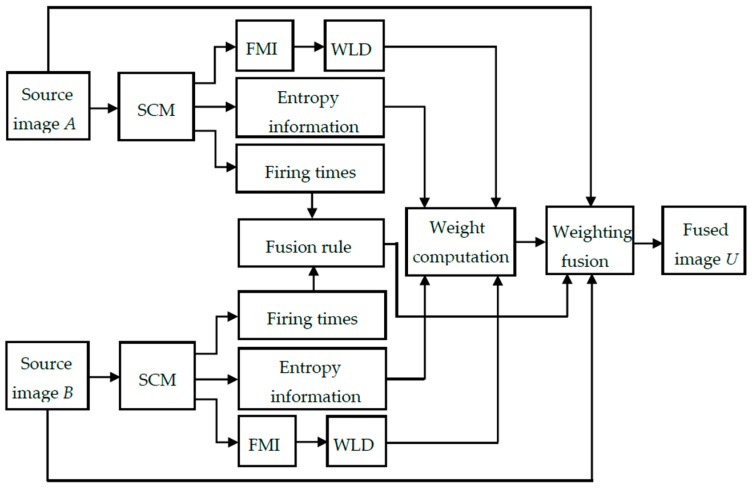
The flowchart of the SCM-M method.

The weighting fusion framework of the proposed SCM-M method is given in Figure 3. The key components of this method include the fusion rule and the weight computation. In the proposed method, the fusion rule is established based on the firing times of pulse outputs generated by the SCM. The weight is computed based on the similarity between the two source images, which is determined by combining the entropy information of pulse outputs from the SCM with the WLD operating on the resultant firing mapping image (FMI).

### 3.1. Fusion Rule

The two source images, *A* and *B*, are normalized and fed into the two SCMs as the external stimulus. By running the SCMs for *N_max_* times, we will obtain the firing times $T_{i,j}^{A}$ and $T_{i,j}^{B}$ for each pixel at (*i*,*j*) in the source images as:

(5) $$T_{i,j}^{A}=\sum_{k=1}^{N_{\max}}Y_{i,j}^{A}[k]$$

(6) $$T_{i,j}^{B}=\sum_{k=1}^{N_{\max}}Y_{i,j}^{B}[k]$$

**Figure 4:**
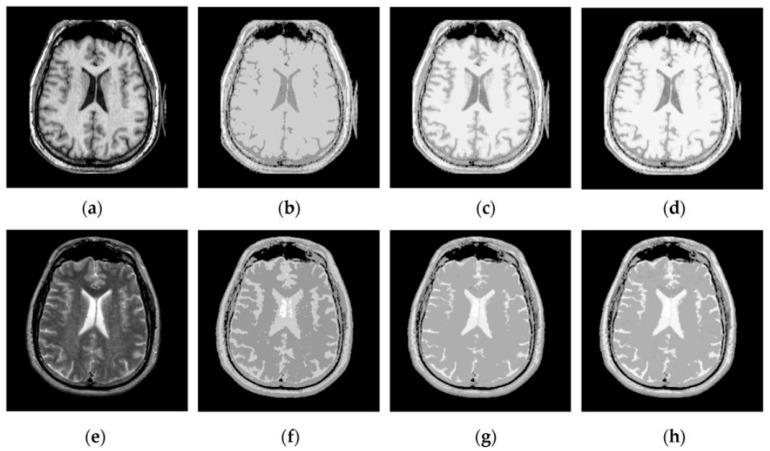
Firing mapping images corresponding to MR-T2 and MR-T1 images: (**a**,**e**) MR-T2 and MR-T1 images; (**b**,**f**) FMIs with *N_max_* = 10 for (a,e); (**c**,**g**) FMIs with *N_max_* = 30 for (a,e); and (**d**,**h**) FMIs with *N_max_* = 50 for (a,e).

Based on the firing times $T_{i,j}^{A}$ and $T_{i,j}^{B}$, two FMIs $T^{A}$ and $T^{B}$ will be produced. From Equations (5) and (6), we can see that the FMI is actually the sum of temporal series of pulse outputs produced by the SCM. Figure 4 shows the FMIs generated by the SCM with *f* = 0.9, *g* = 0.3, *h* = 20 and different *N_max_* operating on a pair of MR images. It should be noted that here the FMIs have been scaled linearly to fit the range [0, 255]. In Figure 4, we can see that the FMI provides a means for representing the information of source images. The representation ability is related to the parameters of the SCM, especially the parameter *N_max_*. A too small (e.g., *N_max_* = 10) or a too large *N_max_* (e.g., *N_max_* = 50) tends to produce the loss of important image details as shown in Figure 4b,d. A proper *N_max_* for the SCM can facilitate representing image details in the source images very well, which is of great significance for medical image fusion.

For each pixel at (*i*,*j*) in two FMIs $T^{A}$ and $T^{B}$, two image patches of size (2*L_p_* + 1) × (2*L_p_* + 1) centered at this pixel will be considered in order to represent the local statistical characteristics, which is more advantageous for the effective fusion of source images than the characteristics of individual pixels. The statistical characteristics of the two image patches will be characterized by the local energy $E_{i,j}^{A}$ and $E_{i,j}^{B}$ defined as:

(7) $$E_{i,j}^{A}=\sqrt{\sum_{m=-L_{p}}^{L_{p}}\sum_{n=-L_{p}}^{L_{p}}(T_{i+m,j+n}^{A}{)}^{2}}$$

(8) $$E_{i,j}^{B}=\sqrt{\sum_{m=-L_{p}}^{L_{p}}\sum_{n=-L_{p}}^{L_{p}}(T_{i+m,j+n}^{B}{)}^{2}}$$

According to the relationship between $E_{i,j}^{A}$ and $E_{i,j}^{B}$, the following fusion rule will be established and correspondingly the intensity of the pixel at (*i*,*j*) in the fused image *U* will be expressed as:

(9) $$U_{i,j}=\left\{ \begin{array}{ll} \omega_{i,j}I_{i,j}^{A}+(1-\omega_{i,j})I_{i,j}^{B} & E_{i,j}^{A}>E_{i,j}^{B}\\ (1-\omega_{i,j})I_{i,j}^{A}+\omega_{i,j}I_{i,j}^{B} & E_{i,j}^{A}\leq E_{i,j}^{B} \end{array}\right.$$

where $I_{i,j}^{A}$ and $I_{i,j}^{B}$ denote the pixel intensity at (*i*,*j*) in the source images *A* and *B*, respectively; $\omega_{i,j}$ denotes the weight, which has an important influence on the quality of the fused image because it will determine the contribution of source images to the fused result. To ensure good image fusion effect, $\omega_{i,j}$ will generally take a relatively big value to highlight the contribution of $I_{i,j}^{A}$ if $E_{i,j}^{A}>E_{i,j}^{B}$, and otherwise it will take a relatively small value to underscore the contribution of $I_{i,j}^{B}$.

### 3.2. Weight Computation

To obtain the suitable weight $\omega_{i,j}$, the local neighborhood (i.e., image patch) $Q_{i,j}$ of size (2*L_p_* + 1) × (2*L_p_* + 1) centered at (*i*,*j*) in any source image will be considered. It is desirable to compute this weight based on the similarity between two image patches $Q_{i,j}^{A}$ and $Q_{i,j}^{B}$. To determine this similarity effectively, the gray-level information and the saliency of $Q_{i,j}$ will be utilized simultaneously. Here, the entropy of pulse outputs and the Weber local descriptor proposed in [42] will be adopted to characterize the gray-level distribution of $Q_{i,j}$ and its saliency, respectively.

#### 3.2.1. Similarity Computation Based on the Entropy Information

For any pixel at (*i*,*j*) in each pulse image at the *n-*th iteration, the image patch $G_{i,j}[n]$ of size (2*L_p_* + 1) × (2*L_p_* + 1) centered at this pixel is considered. To describe the information contained in $G_{i,j}[n]$, its Shannon entropy $H_{i,j}[n]$ is utilized. The entropy $H_{i,j}[n]$ is computed as:

(10) $$H_{i,j}[n]=-P_{i,j}^{1}[n]\log_{2}^{P_{i,j}^{1}[n]}-P_{i,j}^{0}[n]\log_{2}^{P_{i,j}^{0}[n]}$$

where $P_{i,j}^{1}[n]$ and $P_{i,j}^{0}[n]$ $\left(P_{i,j}^{0}[n]=1-P_{i,j}^{1}[n]\right)$ denote the probability of the 1’s and 0’s in $G_{i,j}[n]$, respectively. Here, the probability $P_{i,j}^{1}[n]$ is defined as:

(11) $$P_{i,j}^{1}[n]=\frac{K_{i,j}[n]}{\left(2L_{p}+1)\times (2L_{p}+1\right)}$$

where $K_{i,j}[n]$ denotes the number of 1’s in $G_{i,j}[n]$. 

The Shannon entropy from the various iterations will form the feature vector $V_{i,j}$ ($V_{i,j}=\{H_{i,j}[1],H_{i,j}[2],\ldots ,H_{i,j}[N_{\max}-1],H_{i,j}[N_{\max}]\}$). From Equation (10), we can see that if the image patch $Q_{i,j}$ in any source image is a homogenous region, the Shannon entropy $H_{i,j}[n]$ for all the iterations will be zero because $P_{i,j}^{1}[n]=1$ or $P_{i,j}^{0}[n]=1$, thereby producing a zero vector $V_{i,j}$. Otherwise, because $H_{i,j}[n]$ will not be zero for some iteration times, $V_{i,j}$ will include the nonzero elements, whose values will depend on the gray-level distribution of $Q_{i,j}$. The above analysis indicates that the gray-level information of $Q_{i,j}$ can be characterized by $V_{i,j}$. Accordingly, $V_{i,j}$ can be considered as the feature extracted from $Q_{i,j}$.

The difference $D_{i,j}$ between the features of two image patches $Q_{i,j}^{A}$ and $Q_{i,j}^{B}$ is calculated as:

(12) $$D_{i,j}=||V_{i,j}^{A}-V_{i,j}^{B}|{|}_{2}$$

where $||\cdot |{|}_{2}$ denotes the Euclidean norm. 

Based on the difference $D_{i,j}$, the similarity $S_{i,j}^{En}$ between $Q_{i,j}^{A}$ and $Q_{i,j}^{B}$ based on the entropy information will be defined as:

(13) $$S_{i,j}^{En}=1-\frac{D_{i,j}}{CS_{1}}$$

where $CS_{1}$ is a constant.

#### 3.2.2. Similarity Computation Based on the WLD

In this paper, the WLD is adopted to extract the salient features of an image patch of interest in the firing mapping image, which can be utilized to represent the saliency of the image patch $Q_{i,j}$ in any source image. The WLD is chosen due to its high computational efficiency and excellent ability in finding local salient patterns within an image to simulate the pattern perception of human beings [42]. Indeed, there are many other sparse and dense descriptors such as the scale invariant feature transform (SIFT) and the local binary pattern (LBP). Compared with the SIFT the LBP, the WLD is computed around a relatively small square region and it extracts the local salient patterns by means of the differential excitation [42]. Therefore, the WLD can capture more local salient patterns than the SIFT and the LBP. 

The computation of the WLD stems from Weber’s Law that the ratio of the increment threshold (i.e., a just noticeable difference) to the background intensity is a constant. For the current pixel at (*i*,*j*) in the FMI $T^{A}$ or $T^{B}$, the difference $R_{i,j}$ between this pixel and its neighbors in an image patch of size (2*L_p_* + 1) × (2*L_p_* + 1) is given by:

(14) $$R_{i,j}=\sum_{m=-L_{p}}^{L_{p}}\sum_{n=-L_{p}}^{L_{p}}(T_{i+m,j+n}-T_{i,j})$$

It can be seen from Equation (14) that the computation of $R_{i,j}$ is very similar to the Laplacian operation. Following the hints in Weber’s Law, the differential excitation $\xi_{i,j}$ of the current pixel for the WLD is computed as [42]:

(15) $$\xi_{i,j}=\arctan(\frac{R_{i,j}}{T_{i,j}})=\arctan\left[\sum_{m=-L_{p}}^{L_{p}}\sum_{n=-L_{p}}^{L_{p}}\left(\frac{T_{i+m,j+n}-T_{i,j}}{T_{i,j}}\right)\right]$$

where the arctangent function is used to prevent the output from increasing or decreasing too quickly when the input becomes larger or smaller [42]. 

**Figure 5:**
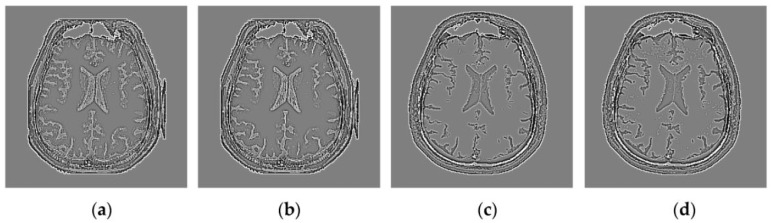
The Weber local descriptor (WLD) for the firing mapping images (FMIs) shown in Figure 4: (**a**) the WLD for Figure 4c; (**b**) the WLD for Figure 4d; (**c**) the WLD for Figure 4g; and (**d**) the WLD for Figure 4h.

As discussed in [42], the WLD can indicate the saliency of the local neighborhood very well because of its powerful representation ability for such important features as edges and textures. To explain this point better, Figure 5 shows the results of the WLD operating on the FMIs shown in Figure 4c,d,g,h. The comparisons between Figure 4 and Figure 5 show that both the strong and weak edges in Figure 4 have become more salient in the results of the WLD than in the FMIs. Therefore, the WLD operating on the FMIs can bring out the local image structural features of source images very well, which are highly beneficial for medical diagnosis based on different imaging modalities. It will be desirable to utilize these extracted salient image features to determine the similarity $S_{i,j}^{WLD}$ between $Q_{i,j}^{A}$ and $Q_{i,j}^{B}$ in the source images, i.e.,

(16) $$S_{i,j}^{WLD}=1-\frac{\left|\xi_{i,j}^{A}-\xi_{i,j}^{B}\right|}{CS_{2}}$$

where $CS_{2}$ is a constant.

#### 3.2.3. Weight Determining Based on the Combined Similarity

By combining $S_{i,j}^{En}$ with $S_{i,j}^{WLD}$, the weight $\omega_{i,j}$ in Equation (9) will be presented as:

(17) $$\omega_{i,j}=S_{i,j}^{En}\cdot S_{i,j}^{WLD}$$

From Equation (17), we can see that if the two image patches centered at (*i*,*j*) in the source images have the same local structure, which means the same WLD, $\omega_{i,j}$ will be determined by $S_{i,j}^{En}$ which is related to the intensity distributions of image patches in the source images. Likewise, $\omega_{i,j}$ will depend on $S_{i,j}^{WLD}$, which is related to the local image structure if the two image patches have the same gray-level distributions. The above analysis shows that $\omega_{i,j}$ can represent the similarity between two image patches effectively by combining their gray-level information with their local structure. It should be noted that $\omega_{i,j}$ is also likely to be computed using such non-Euclidean similarity measures as Cosine distance measure and Pearson correlation, which is scale and translation invariant. When Pearson correlation is used to measure the similarity between Shannon entropy feature vectors of two considered image patches, it can address scale and translation changes of feature vectors. However, this correlation requires that the variables follow a bivariate normal distribution. The possibility of utilizing non-Euclidean similarity measures for the similarity computation in the weighting fusion strategy will be explored in-depth in future work.

### 3.3. Implementation of the SCM-M Method

The implementation of the proposed SCM-M method can be summarized as the following steps:

(1) The two source images *A* and *B* are input into two SCMs. After running the SCM for *N_max_* times, the series of binary pulse images will be obtained for the source images using Equations (1)–(4).
(2) For each pixel at (*i*,*j*) in *A* and *B*, the Shannon entropy from the various iterations is computed on the output pulse images using Equation (10) to generate two feature vectors $V_{i,j}^{A}$ and $V_{i,j}^{B}$. Based on the difference between the two feature vectors, the similarity $S_{i,j}^{En}$ between two image patches $Q_{i,j}^{A}$ and $Q_{i,j}^{B}$ centered at (*i*,*j*) is computed using Equation (13).
(3) The output pulse images are utilized to generate the firing mapping images for two source images. For any pixel at (*i*,*j*) in two FMIs, the local energy $E_{i,j}^{A}$ and $E_{i,j}^{B}$ are computed on the considered two image patches centered at this pixel using Equations (7) and (8), respectively. Meanwhile, the WLD is computed for the two image patches using Equation (15) to determine the similarity $S_{i,j}^{WLD}$ between $Q_{i,j}^{A}$ and $Q_{i,j}^{B}$ using Equation (16).
(4) The weight $\omega_{i,j}$ is determined by $S_{i,j}^{En}$ and $S_{i,j}^{WLD}$ using Equation (17).
(5) According to the relationship between $E_{i,j}^{A}$ and $E_{i,j}^{B}$, the fused image is produced by the weighted sum of two source images using Equation (9).

## 4. Experimental Results and Discussions

**Figure 6:**
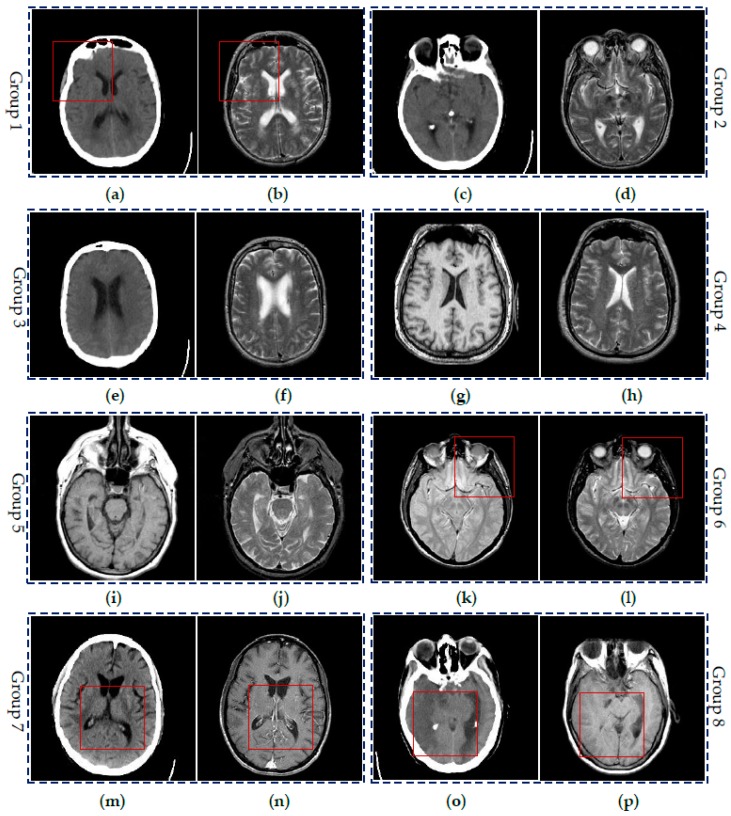
Six groups of source medical images: (**a**,**c**,**e**,**m**,**o**) CT images; (**b**,**d**,**f**,**h**,**j**,**l**) MR-T2 images; (**g**,**i**,**p**) MR-T1 images; (**k**) MR-PD image; and (**n**) MR-GAD image.

To demonstrate the effectiveness of the proposed SCM-M method, extensive experiments have been done on eight groups of CT and MR images shown in Figure 6. All the images are chosen from the website [43]. Each image is of size 256 × 256. Two images in each image pair include the complementary information. Here, Groups 1–3 are three pairs of CT and MR images of different regions in the brain of a patient with acute stroke. Group 4 includes the transaxial MR images of the normal brain. Groups 5 and 6 are MR images of the brain of patients with vascular dementia and AIDS dementia, respectively. Groups 7 and 8 are two pairs of CT and MR images of the brain of the patients with cerebral toxoplasmosis and fatal stroke. Please note that intensity standardization and inhomogeneity correction have been performed on all MR images by the above website. 

To verify the advantage of the SCM-M method, it has been compared with the discrete wavelet transform (DWT), the NSCT [21], the combination of NSCT and sparse representation (NSCT-SR) [21], m-PCNN [29], PCNN-NSCT [32] and SCM-F [41] based fusion methods. The code of the DWT and PCNN-NSCT methods is available on the websites [44,45], respectively. The code of the NSCT and NSCT-SR methods can be found on the website [46].

### 4.1. Parameter Settings

For the DWT fusion method, the wavelet and the number of decomposition levels are chosen to be DBSS(2,2) and 4, respectively. For the m-PCNN method and the SCM-F method, all the involved parameters are chosen as suggested in [29,41], respectively. For the NSCT and NSCT-SR methods, we have used the “pyrexc” as the pyramid filter and the “vk” as the directional filter. The number of directions of the four decomposition levels from coarse to fine is selected as 2, 3, 3, 4, respectively. For the PCNN-NSCT method, we have chosen the decay constant $\alpha_{L}=0.5$, the linking strength β=3 and the amplitude gain $V_{\theta }=0.5$ in the PCNN while keeping other parameters to be the same as those in [32]. As regards the proposed method, we have fixed *f* = 0.9, *g* = 0.3, *h* = 20, $L_{p}=1$, *CS*_1_ = 3 × *N*_max_, *CS*_2_ = 5 × π and chosen *N*_max_ to be close to 20. 

### 4.2. Visual Comparisons of Fused Results

**Figure 7:**
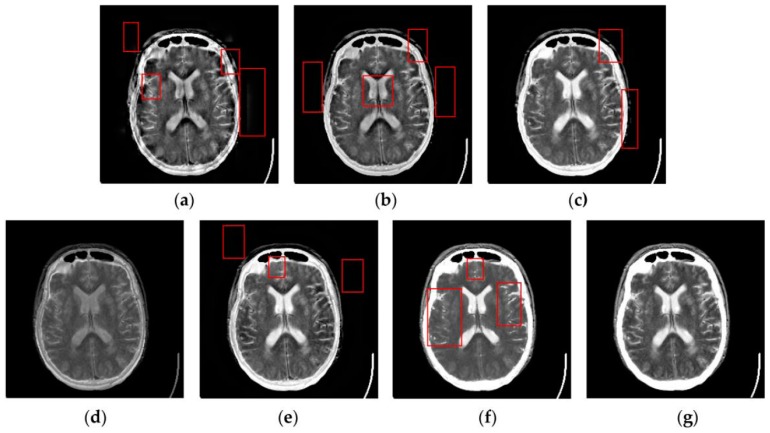
Fused results of the evaluated methods for the first group of source images shown in Figure 6a,b: (**a**) the discrete wavelet transform (DWT) method; (**b**) the non-subsampled contourlet transform (NSCT) method; (**c**) the NSCT-SR method; (**d**) the multi-channel pulse coupled neural network (m-PCNN) method; (**e**) the PCNN-NSCT method; (**f**) the SCM-F method; and (**g**) the SCM-M method.

**Figure 8:**
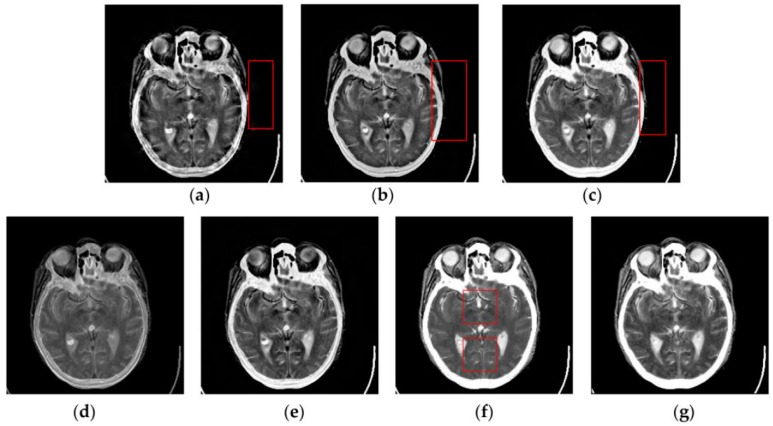
Fused results of the evaluated methods for the second group of source images shown in Figure 6c,d: (**a**) the DWT method; (**b**) the NSCT method; (**c**) the NSCT-SR method; (**d**) the m-PCNN method; (**e**) the PCNN-NSCT method; (**f**) the SCM-F method; and (**g**) the SCM-M method.

**Figure 9:**
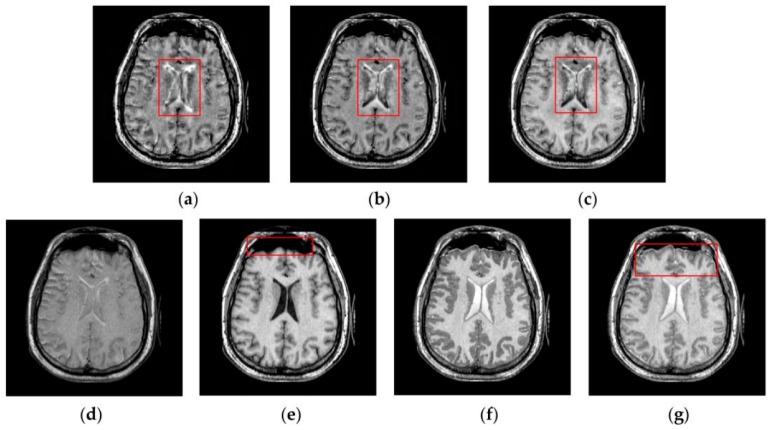
Fused results of the evaluated methods for the fourth group of source images shown in Figure 6g,h: (**a**) the DWT method; (**b**) the NSCT method; (c) the NSCT-SR method; (**d**) the m-PCNN method; (**e**) the PCNN-NSCT method; (**f**) the SCM-F method; and (**g**) the SCM-M method.

**Figure 10:**
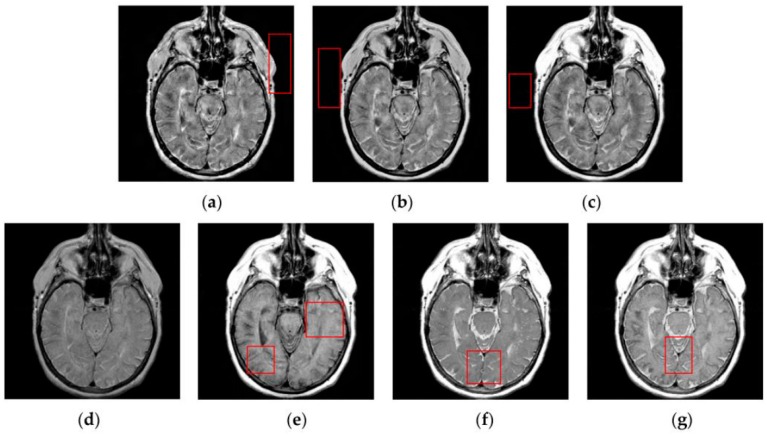
Fused results of the evaluated methods for the fifth group of source images shown in Figure 6i,j: (**a**) the DWT method; (**b**) the NSCT method; (**c**) the NSCT-SR method; (**d**) the m-PCNN method; (**e**) the PCNN-NSCT method; (**f**) the SCM-F method; and (**g**) the SCM-M method.

Figure 7, Figure 8, Figure 9 and Figure 10 show the fused results for the evaluated seven methods operating on such medical image pairs as Groups 1, 2, 4 and 5 shown in Figure 6, respectively. The observation from Figure 7, Figure 8 and Figure 10 shows that the DWT, NSCT and NSCT-SR methods introduce artifacts as well as false information in the fused results as indicated by the red boxes, which will greatly influence the quality of the fused images. Meanwhile, it is shown in Figure 7 and Figure 9 that the above three fusion methods cannot preserve image details well in that they produce the obvious distortion of image details marked by the red boxes in the fused results. The m-PCNN method cannot maintain the luminance of the fused results and it produces such low-contrast fused images that some important image details are difficult to identify, which is very disadvantageous for clinical diagnosis. The PCNN-NSCT method and the SCM-F method lead to loss of some important details in the source images to different extent. For example, for Groups 4 and 5, although almost all the details in the MR-T1 images can be transferred to the fused images by the PCNN-NSCT method very well, many details in the MR-T2 images have not been preserved by this method as indicated by the red boxes in the fused images shown in Figure 9e and Figure 10e. For Groups 1, 2, and 5, some image details have been seriously damaged by the SCM-F method as shown by the red boxes in Figure 7f, Figure 8f and Figure 10f. 

By comparison, the SCM-M method not only provides high contrast for the fused images, but also maintains important information from the various source images in the fused results effectively. In particular, the proposed method can preserve fine image details very well as shown by the red boxes in Figure 9g and Figure 10g without introducing artifacts or leading to edge blurring. The above comparisons demonstrate the superiority of the SCM-M method over other compared methods in that the fused images obtained by this method are more clear, informative, and have higher contrast. 

**Figure 11:**
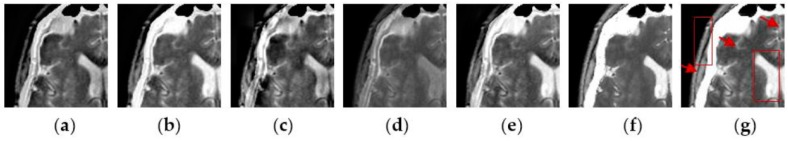
Enlarged views of fused results of ROIs denoted by the red boxes in Group 1 in Figure 6 for the seven methods: (**a**) the DWT method; (**b**) the NSCT method; (**c**) the NSCT-SR method; (**d**) the m-PCNN method; (**e**) the PCNN-NSCT method; (**f**) the SCM-F method; and (**g**) the SCM-M method.

**Figure 12:**
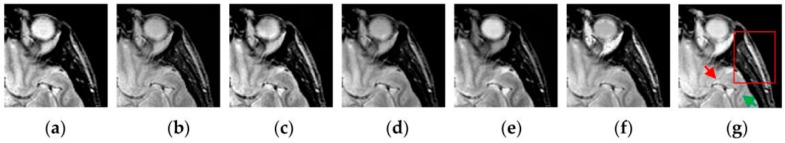
Enlarged views of fused results of ROIs denoted by the red boxes in Group 6 in Figure 6 for the seven methods: (**a**) the DWT method; (**b**) the NSCT method; (**c**) the NSCT-SR method; (**d**) the m-PCNN method; (**e**) the PCNN-NSCT method; (**f**) the SCM-F method; and (**g**) the SCM-M method.

**Figure 13:**
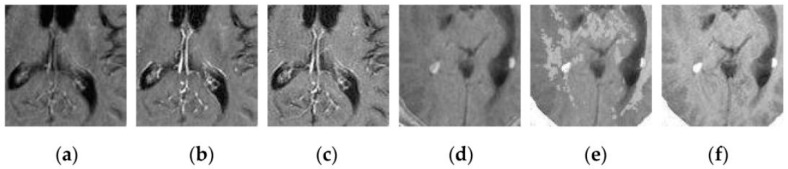
Enlarged views of fused results of ROIs denoted by the red boxes in Groups 7 and 8 shown in Figure 6 for the m-PCNN, SCM-F and SCM-M methods: (**a**) the m-PCNN method for Group 7; (**b**) the SCM-F method for Group 7; (**c**) the SCM-M method for Group 7; (**d**) the m-PCNN method for Group 8; (**e**) the SCM-F method for Group 8; and (**f**) the SCM-M method for Group 8.

To further verify the advantage of the proposed SCM-M method in multimodal image fusion, Figure 11 and Figure 12 show the enlarged views of fused results for all evaluated methods operating on regions of interest (ROIs) denoted by the red boxes in Groups 1 and 6 in Figure 6, respectively. Figure 13 shows the enlarged views of fused results for the proposed method, the m-PCNN method and the SCM-F method operating on ROIs denoted by the red boxes in Groups 7 and 8 shown in Figure 6. In Figure 11 and Figure 12, we can see that the SCM-M method can maintain the salient information in the source images and provide better visual perception with less loss in luminance or contrast than other compared methods. To explain this point better, some edges and regions have been chosen from Figure 11g and Figure 12g. It can be seen from Figure 11 that the SCM-M method can provide better edge preservation than all other methods as pointed by the three red arrows. Meanwhile, compared with the DWT, NSCT and NSCT-SR methods, the SCM-M method can maintain the information in the MR image shown in Figure 6f better without introducing artifacts as indicated by the two red boxes. In Figure 12, we can see that the proposed method can keep the integrity of the edge marked by the red arrow best among all evaluated methods. Likewise, as pointed by the green arrow, the edge can be preserved very well by the proposed method while it has been damaged very seriously by other methods. Besides, the sharpness of the region shown by the red box can be maintained by the proposed method better than by the compared method. Furthermore, it can be seen in Figure 13 that compared with the m-PCNN and SCM-F methods, the SCM-M method can preserve fine image details and maintain image contrast better.

### 4.3. Quantitative Comparison of Fused Results

The performance of these compared methods is appreciated in terms of quantitative indexes including mutual information ($Q_{M}$) [47], edge preservation index ($Q_{E}$) [48], structural similarity (SSIM) [49] based metric ($Q_{S}$) [50], fusion quality index ($Q_{L}$) [51] and the fusion similarity metric ($Q_{T}$) [52] and standard deviation (*STD*). Higher values for these indexes indicate better fusion results. 

#### (1) $Q_{M}$

The metric $Q_{M}$ reflects the total amount of information that the fused image contains about two source images, and it is defined as:

(18) $$Q_{M}=\mathrm{MI}(A,U)+\mathrm{MI}(B,U)$$

where

(19) $$\mathrm{MI}(A,U)=\sum_{a\in A}\sum_{u\in U}p(a,u)\log_{2}\frac{p(a,u)}{p(a)p(u)}$$

(20) $$\mathrm{MI}(B,U)=\sum_{b\in B}\sum_{u\in U}p(b,u)\log_{2}\frac{p(b,u)}{p(b)p(u)}$$

where p(a,u) and p(b,u) are the discrete joint probability, p(b) and p(u) are the marginal discrete probabilities of *A*, *B* and U and are obtained by summing *p* over *a*, *b*, and *u*, respectively.

#### (2) $Q_{E}$

The metric $Q_{E}$ measures the similarity between the edges transferred during the fusion process, and it is defined as:

(21) $$Q_{E}=\frac{\sum_{m=1}^{Y}\sum_{n=1}^{Z}Q_{m,n}^{A,U}w_{m,n}^{A}+Q_{m,n}^{B,U}w_{m,n}^{B}}{\sum_{m=1}^{Y}\sum_{n=1}^{Z}w_{m,n}^{A}+w_{m,n}^{B}}$$

where

(22) $$Q_{m,n}^{A,U}=Q_{g,m,n}^{A,U}Q_{\alpha ,m,n}^{A,U}$$

(23) $$Q_{m,n}^{B,U}=Q_{g,m,n}^{B,U}Q_{\alpha ,m,n}^{B,U}$$

where $Q_{g,m,n}^{*,U}$ and $Q_{\alpha ,m,n}^{*,U}$ denote the edge strength and orientation preservation values at (*m*, *n*) in the image *A* or *B*, respectively; $w_{m,n}^{A}$ and $w_{m,n}^{B}$ denotes the weight for $Q_{m,n}^{A,U}$ and $Q_{m,n}^{B,U}$, respectively; and Y and Z are the width and the height of U, respectively.

#### (3) $Q_{S}$

The metric $Q_{S}$ employs the local SSIM between the source images as a match measure, according to which different operations are applied to the evaluations of different local regions [50]. This metric is defined as:

(24) $$Q_{S}=\frac{1}{\left|W_{s}\right|}\sum_{w_{s}\in W_{s}}Q\left(A,B,U|w_{s}\right)$$

where

(25) $$Q\left(A,B,U|w_{s}\right)=\left\{ \begin{array}{ll} \lambda \left(w_{s}\right)\mathrm{SSIM}\left(A,U|w_{s}\right)+\left(1-\lambda \left(w_{s}\right)\right)\mathrm{SSIM}\left(B,U|w_{s}\right) & \mathrm{SSIM}\left(A,B|w_{s}\right)\geq 0.75\\ \max\left\{\mathrm{SSIM}\left(A,U|w_{s}\right),\mathrm{SSIM}\left(B,U|w_{s}\right)\right\} & \mathrm{SSIM}\left(A,B|w_{s}\right)<0.75 \end{array}\right.$$

where $w_{s}$ is a sliding window, $\lambda \left(w_{s}\right)$ is the local weight, $W_{s}$ is the family of all sliding windows, $\left|W_{s}\right|$ is the cardinality of $W_{s}$, $\mathrm{SSIM}\left(A,U{|w}_{s}\right)$ is a measure for the similarity between the sliding window $w_{s}$ in *A* and that in U, and a similar definition can be extended to $\mathrm{SSIM}\left(B,U{|w}_{s}\right)$ and $\mathrm{SSIM}\left(A,B|w_{s}\right)$. For the computation of $Q_{S}$, all the involved parameters are kept to be same to those in [50] except that two constants *C*_1_ and *C*_2_ are chosen to be 2 × 10^−6^ for the computation of SSIM. 

#### (4) $Q_{L}$*s*

The fusion quality index $Q_{L}$ is computed as:

(26) $$Q_{L}=Q\left(A,B,U|w_{s}\right)\cdot Q\left(A^{\prime },B^{\prime },U^{\prime }|w_{s}\right)$$

where $A^{\prime }$, $B^{\prime }$ and $U^{\prime }$ denote the edge images of *A*, *B* and *U*, respectively. $Q\left(A,B,U|w_{s}\right)$ is defined as:

(27) $$Q\left(A,B,U|w_{s}\right)=\frac{\sum_{w_{s}\in W_{s}}C(w_{s})\left(\lambda \left(w_{s}\right)\mathrm{SSIM}\left(A,U|w_{s}\right)+\left(1-\lambda \left(w_{s}\right)\right)\mathrm{SSIM}\left(B,U|w_{s}\right)\right)}{\sum_{w_{s}\in W_{s}}C(w_{s})}$$

where $C(w_{s})$ represents the overall saliency of the sliding window $w_{s}$ and it is chosen as $C(w_{s})=\max(s(A|w_{s})+s_{\max},s(B|w_{s})+s_{\max})$ with $s(A|w_{s})$, $s(B|w_{s})$ and $s_{\max}$ denoting the variance of the window $w_{s}$ in the image *A* and that in the image *B*, and the difference between the maximum variance of all sliding windows in *A* and that in *B*, respectively.

#### (5) $Q_{T}$

The fusions similarity index $Q_{T}$ is computed as:

(28) $$Q_{T}=\sum_{w_{s}\in W_{s}}sim\left(A,B,U|w_{s}\right)\cdot Q\left(A,U|w_{s}\right)+\left(1-sim\left(A,B,U|w_{s}\right)\right)\cdot Q\left(B,U|w_{s}\right)$$

where $Q\left(A,U|w_{s}\right)$ and $Q\left(B,U|w_{s}\right)$ are computed based on the universal image quality index [53]; and $sim\left(A,B,U|w_{s}\right)$ is dependent on the similarity in spatial domain between the input images and the fused image and it is defined as a piecewise function presented in [52].

#### (6) STD

The metric STD can measure the contrast of the fused image, and it is defined as:

(29) $$STD=\sqrt{\frac{\sum_{i=1}^{Y}\sum_{j=1}^{Z}{\left(U_{i,j}-\bar{U}\right)}^{2}}{\left(Y\cdot Z-1\right)}}$$

where $\bar{U}$ is the mean intensity of the fused image.

Table 1, Table 2, Table 3, Table 4, Table 5 and Table 6 list $Q_{M}$, $Q_{E}$, $Q_{S}$, $Q_{L}$, $Q_{M}$ and STD of fused results for seven evaluated methods operating on the six groups of multi-modal medical images, respectively. In these tables, the “bold” value denotes the highest one for each metric. From these Tables, we can see that among all evaluated methods, the DWT produces the lowest $Q_{M}$ values and the NSCT method provides the smallest $Q_{S}$ and $Q_{T}$ for all test images. For the majority of medical image pairs, the m-PCNN method provides the lowest $Q_{E}$ and STD values while the PCNN-NSCT method produces the lowest $Q_{L}$ values. Compared with other evaluated methods, the SCM-M method provides higher six metrics values in all cases except that it is outperformed by the NSCT and NSCT-SR methods in terms of $Q_{E}$ for Groups 7 and 8 and the SCM-F method in terms of $Q_{L}$ for Group 6. The above comparisons demonstrate the superiority of the proposed method over the compared fusion methods in maintaining the information of source images, preserving the local image structure and image details, and ensuring the contrast of the fused image.

To further demonstrate the superiority of the proposed method over other compared methods, the paired *t*-tests have been performed based on the data in Table 1, Table 2, Table 3, Table 4, Table 5 and Table 6. The test results are listed in Table 7. The *p* values in Table 7 indicate that there exists very significant difference between the proposed method and other evaluated methods in terms of mutual information, structural similarity based metric, fusion similarity metric and standard deviation. As regards edge preservation index, there is no significant difference between the proposed method and the NSCT and NSCT-SR methods, but there still exists the significant difference between the proposed method and the DWT, PCNN-NSCT, and SCM-F methods. As for fusion quality index, the difference between the SCM-M method and the NSCT-SR and SCM-F methods is significant while the difference between the proposed method and the remaining four methods is very significant. 

Here we will make a simple analysis of the reason why the proposed method generally outperforms the m-PCNN method and the SCM-F method, which are similar to our method because of the utilization of the third generation neural networks. For the m-PCNN method, the fused image is produced based on the internal activity, which is only related to the pulse output of the individual neuron. In other words, only the individual neurons corresponding to the individual pixels in the two source images are used for image fusion while the characteristics of neighboring neurons of the considered neuron, which can facilitate representing the local image structure, has not been considered in this method. The same problem exists for the SCM-F method, in which only the firing times of the individual neurons is utilized to generate the fused image using the simple choosing and averaging strategy. By comparison, in the proposed SCM-M method, the characteristics of neurons in a local neighborhood are considered for the construction of the fusion rule and the determining of the fusion weight. As regards the fusion rule, the firing times of all the neurons in a neighborhood will be a more effective metric for the establishment of the fusion rule than that of the individual neuron. For the fusion weight, the WLD operating on the firing mapping image and the entropy information of pulse outputs of the SCM are computed in a local neighborhood to produce the weight. The combination of the WLD with the entropy information can facilitate determining the fusion weight effectively in that they can describe the local image structure and the gray-level information of source images very well. 

## 5. Conclusions

A novel spiking cortical model based medical image fusion method is presented in this paper. The proposed method utilizes the pulse outputs of the SCM to realize pixel-level image fusion. By combining the gray-level image information represented by the entropy of pulse outputs with the local image structure represented by the Weber local descriptor operating on the firing mapping image, the proposed method can realize the effective weighted fusion of source images. Extensive experiments on the various CT and MR images demonstrate that the proposed method can produce clearer, more informative, higher contrast fused images than numerous existing methods in terms of human vision. Meanwhile, the objective comparison indicates that the proposed method outperforms the compared methods in terms of mutual information, edge preservations metric, structural similarity and standard deviation. Future work will be focused on extending our method to the fusion of multi-spectral medical images such as PET and SPECT images. 

## Figures and Tables

**Table 1:** $Q_{M}$ for the seven methods operating on the eight groups of medical images.

Image Pairs	DWT	NSCT	NSCT-SR	m-PCNN	PCNN-NSCT	SCM-F	SCM-M
Group 1	2.7762	3.0183	3.3993	3.7837	3.1382	4.0495	**5.0609**
Group 2	2.7632	3.0260	3.2706	3.9376	3.0969	4.2426	**5.1302**
Group 3	2.7708	3.0362	3.3459	3.6843	3.2681	3.9568	**4.9279**
Group 4	2.5753	2.8873	3.1128	3.6600	4.6146	4.6320	**5.4548**
Group 5	3.0404	3.1078	3.4823	3.8007	3.8178	4.9843	**6.1422**
Group 6	3.4840	3.7787	3.5974	3.9726	3.9684	4.2431	**5.5556**
Group 7	2.9382	3.1397	3.2482	3.5257	3.2624	3.9406	**5.3860**
Group 8	2.8822	3.0045	3.0446	3.7121	3.0854	4.2942	**5.0863**

**Table 2:** $Q_{E}$ for the seven methods operating on the eight groups of medical images.

Image Pairs	DWT	NSCT	NSCT-SR	m-PCNN	PCNN-NSCT	SCM-F	SCM-M
Group 1	0.5131	0.6044	0.6256	0.3416	0.5750	0.5344	**0.6357**
Group 2	0.5049	0.5972	0.6127	0.3662	0.5464	0.5378	**0.6224**
Group 3	0.4753	0.5482	0.5772	0.3173	0.5255	0.4919	**0.5867**
Group 4	0.4569	0.5704	0.5741	0.3929	0.6579	0.5461	**0.6298**
Group 5	0.4544	0.5592	0.5606	0.3366	0.4815	0.5193	**0.5937**
Group 6	0.6584	**0.7048**	0.7010	0.6974	0.6679	0.6639	0.7045
Group 7	0.4834	**0.5676**	0.5653	0.4174	0.5030	0.4770	0.5503
Group 8	0.4383	**0.5651**	0.5634	0.3627	0.4878	0.4587	0.5104

**Table 3:** $Q_{S}$ for the seven methods operating on the eight groups of medical images.

Image Pairs	DWT	NSCT	NSCT-SR	m-PCNN	PCNN-NSCT	SCM-F	SCM-M
Group 1	0.6369	0.4501	0.5484	0.7877	0.6017	0.9081	**0.9671**
Group 2	0.6550	0.4551	0.5374	0.7772	0.5432	0.9077	**0.9542**
Group 3	0.6264	0.4510	0.5316	0.7887	0.5874	0.9089	**0.9693**
Group 4	0.6515	0.5172	0.6109	0.7586	0.8935	0.9118	**0.9467**
Group 5	0.6401	0.5221	0.6423	0.6717	0.7193	0.8645	**0.9234**
Group 6	0.7543	0.6456	0.6612	0.9170	0.6405	0.9347	**0.9753**
Group 7	0.6426	0.4753	0.6007	0.8028	0.5866	0.8790	**0.9500**
Group 8	0.6250	0.4473	0.5053	0.7742	0.5958	0.8524	**0.9315**

**Table 4:** $Q_{L}$ for the seven methods operating on the eight groups of medical images.

Image Pairs	DWT	NSCT	NSCT-SR	m-PCNN	PCNN-NSCT	SCM-F	SCM-M
Group 1	0.3333	0.4217	0.5045	0.4913	0.2817	0.6523	**0.7323**
Group 2	0.3533	0.4551	0.5098	0.4724	0.2378	0.6271	**0.6931**
Group 3	0.3138	0.3937	0.4628	0.4834	0.2676	0.6405	**0.7156**
Group 4	0.3006	0.4366	0.4797	0.4420	0.4479	0.5357	**0.5854**
Group 5	0.3280	0.5095	0.5723	0.2928	0.3018	0.4922	**0.5096**
Group 6	0.5355	0.6659	0.7189	0.7681	0.3318	**0.7845**	0.7719
Group 7	0.3290	0.4314	0.5102	0.5183	0.2677	0.5685	**0.5700**
Group 8	0.2972	0.3684	0.3935	0.4488	0.2754	0.4622	**0.5142**

**Table 5:** $Q_{T}$ for the seven methods operating on the eight groups of medical images.

Image Pairs	DWT	NSCT	NSCT-SR	m-PCNN	PCNN-NSCT	SCM-F	SCM-M
Group 1	0.6100	0.4164	0.5159	0.7729	0.5640	0.8734	**0.9337**
Group 2	0.6254	0.4160	0.5023	0.7590	0.5177	0.8672	**0.9163**
Group 3	0.5984	0.4199	0.4988	0.7750	0.5645	0.8745	**0.9351**
Group 4	0.6155	0.4740	0.5689	0.7390	0.8404	0.8628	**0.8991**
Group 5	0.5935	0.4608	0.5778	0.6417	0.6516	0.7996	**0.8515**
Group 6	0.7240	0.4541	0.6085	0.9022	0.5810	0.9075	**0.9135**
Group 7	0.6042	0.4343	0.5587	0.7853	0.5496	0.8355	**0.8952**
Group 8	0.5928	0.4058	0.4662	0.7575	0.5628	0.7981	**0.8773**

**Table 6:** STD for the seven methods operating on the eight groups of medical images.

Image Pairs	DWT	NSCT	NSCT-SR	m-PCNN	PCNN-NSCT	SCM-F	SCM-M
Group 1	66.6193	66.1906	79.5626	52.5581	74.3848	79.8346	**81.0782**
Group 2	67.9753	69.5841	82.3969	55.5187	74.2025	82.5708	**83.7441**
Group 3	64.6307	67.8026	79.8704	49.3479	74.1064	79.7935	**80.8798**
Group 4	69.9537	70.2955	79.6350	64.9548	82.0193	82.5526	**85.1864**
Group 5	73.3412	74.7206	85.5797	57.8297	85.0754	86.0308	**88.7634**
Group 6	72.6172	73.3028	73.2137	64.9728	64.6814	72.4982	**80.1262**
Group 7	68.7288	70.7589	79.8773	55.9421	74.2269	79.7173	**82.8038**
Group 8	79.2868	81.8019	88.7306	64.8613	75.6456	93.8168	**96.9955**

**Table 7:** Paired *t*-test results for the compared methods operating on the eight groups of medical images.

Metrics	DWT	NSCT	NSCT-SR	m-PCNN	PCNN-NSCT	SCM-F
$Q_{M}$	*p* < 0.01	*p* < 0.01	*p* < 0.01	*p* < 0.01	*p* < 0.01	*p* < 0.01
$Q_{E}$	*p* < 0.01	*p >* 0.05	*p >* 0.05	*p* < 0.01	*p* < 0.01	*p* < 0.01
$Q_{S}$	*p* < 0.01	*p* < 0.01	*p* < 0.01	*p* < 0.01	*p* < 0.01	*p* < 0.01
$Q_{L}$	*p* < 0.01	*p* < 0.01	0.01 < *p* < 0.05	*p* < 0.01	*p* < 0.01	0.01 < *p* < 0.05
$Q_{T}$	*p* < 0.01	*p* < 0.01	*p* < 0.01	*p* < 0.01	*p* < 0.01	*p* < 0.01
STD	*p* < 0.01	*p* < 0.01	*p* < 0.01	*p* < 0.01	*p* < 0.01	*p* < 0.01

## References

[1] Kaplan I., Oldenburg N.E., Meskell P., Blake M., Church P., Holupka E.J. (2002). Real time MRI-ultrasound image guided stereotactic prostate biopsy. Magn. Reson. Imaging.

[2] Buck A.K., Herrmann K., Schreyögg J. (2011). PET/CT for staging lung cancer: Costly or cost-saving?. Eur. J. Nucl. Med. Mol. Imaging.

[3] Barra V., Boire J.Y. (2001). A general framework for the fusion of anatomical and functional medical images. NeuroImage.

[4] Van der Ploeg I.M.C., Valdés Olmos R.A., Nieweg O.E., Rutgers E.J.T., Kroon B.B.R., Hoefnagel C.A. (2007). The additional value of SPECT/CT in lymphatic mapping in breast cancer and melanoma. J. Nucl. Med..

[5] Redondo R., Sroubek F., Fischer S., Cristoba G. (2009). Multifocus image fusion using the log-Gabor transform and a multisize windows technique. Inf. Fusion.

[6] Tu T.M., Su S.C., Shyu H.C., Huang P.S. (2001). A new look at IHS-like image fusion methods. Inf. Fusion.

[7] Daneshvar S., Ghassemian H. (2010). MRI and PET image fusion by combining IHS and retina-inspired models. Inf. Fusion.

[8] Wang H.Q., Xing H. Multi-mode medical image fusion algorithm based on principal component analysis. Proceedings of the International Symposium on Computer Network and Multimedia Technology.

[9] Toet A., van Ruyven J.J., Valeton J.M. (1989). Merging thermal and visual images by a contrast pyramid. Opt. Eng..

[10] Petrovic V.S., Xydeas C.S. (2004). Gradient-based multiresolution image fusion. IEEE Trans. Image Process..

[11] Ehsan S., Abdullah S.M.U., Akhtar M.J., Mandic D.P., McDonald-Maier K.D. (2015). Multi-scale pixel-based image fusion using multivariate empirical mode decomposition. Sensors.

[12] Qu G., Zhang D., Yan P. (2001). Medical image fusion by wavelet transform modulus maxima. Opt. Express.

[13] Yang Y., Park D.S., Huang S., Rao N. (2010). Medical image fusion via an effective wavelet-based approach. EURASIP J. Adv. Signal Process..

[14] Singh R., Khare A. (2014). Fusion of multimodal medical images using Daubechies complex wavelet transform—A multiresolution approach. Inf. Fusion.

[15] Ali F.E., El-Dokany I.M., Saad A.A., Abd El-Samie F.E. (2008). Curvelet fusion of MR and CT images. Prog. Electromagn. Res. C.

[16] Das S., Chowdhury M., Kundu M.K. (2011). Medical image fusion based on ripplet transform type-I. Prog. Electromagn. Res. B.

[17] Yang L., Guo B.L., Ni W. (2008). Multimodality medical image fusion based on multiscale geometric analysis of contourlet transform. Neurocomputing.

[18] Li T., Wang Y. (2011). Biological image fusion using a NSCT based variable-weight method. Inf. Fusion.

[19] Liu C., Chen S., Fu Q. (2013). Multi-modality image fusion using the nonsubsampled contourlet transform. IEICE Trans. Inf. Syst..

[20] Bhatnagar G., Wu Q.M.J., Liu Z. (2013). Directive contrast based multimodal medical image fusion in NSCT domain. IEEE Trans. Multimed..

[21] Liu Y., Liu S., Wang Z. (2015). A general framework for image fusion based on multi-scale transform and sparse representation. Inf. Fusion.

[22] Wang J., Lai S., Li M. (2012). Improved image fusion method based on NSCT and accelerated NMF. Sensors.

[23] Wang L., Li B., Tian L. (2014). Multi-modal medical image fusion using the inter-scale and intra-scale dependencies between image shift-invariant shearlet coefficients. Inf. Fusion.

[24] Wang L., Li B., Tian L. (2014). EGGDD: An explicit dependency model for multi-modal medical image fusion in shift-invariant shearlet transform domain. Inf. Fusion.

[25] Jiang H., Tian Y. (2011). Fuzzy image fusion based on modified self-generating neural network. Expert Syst. Appl..

[26] Eckhorn R., Reitboeck H.J., Arndt M., Dicke P.W. (1990). Feature linking via synchronization among distributed assemblies: Simulation of results from cat cortex. Neural Comput..

[27] Eckhorn R., Frien A., Bauer R., Woelbern T., Kehr H. (1993). High frequency (60–90 Hz) oscillations in primary visual cortex of awake monkey. Neuroreport.

[28] Li M., Cai W., Tan Z. (2006). A region-based multi-sensor image fusion scheme using pulse coupled neural network. Pattern Recognit. Lett..

[29] Wang Z., Ma Y. (2008). Medical image fusion using m-PCNN. Inf. Fusion.

[30] Zhao Y., Zhao Q., Hao A. (2014). Multimodal medical image fusion using improved multi-channel PCNN. Bio-Med. Mater. Eng..

[31] Chai Y., Li H.F., Qu J.F. (2010). Image fusion scheme using a novel dual-channel PCNN in lifting stationary wavelet domain. Opt. Commun..

[32] Qu X., Yan J., Xiao H., Zhu Z. (2008). Image fusion algorithm based on spatial frequency-motivated pulse coupled neural networks in nonsubsampled contourlet transform domain. Acta Autom. Sin..

[33] Kong W.W., Lei Y.J., Lei Y., Lu S. (2011). Image fusion technique based on non-subsampled contourlet transform and adaptive unit-fast-linking pulse-coupled neural network. IET Image Process..

[34] El-taweel G.S., Helmy A.K. (2013). Image fusion scheme based on modified dual pulse coupled neural network. IET Image Process..

[35] Das S., Kundu M.K. (2012). NSCT-based multimodal medical image fusion using pulse-coupled neural network and modified spatial frequency. Med. Biol. Eng. Comput..

[36] Das S., Kundu M.K. (2013). A neuro-fuzzy approach for medical image fusion. IEEE Trans. Biomed. Eng..

[37] Ganasala P., Kumar V. (2016). Feature-motivated simplified adaptive PCNN-based medical image fusion algorithm in NSST domain. J. Digit. Imaging.

[38] Geng P., Wang Z., Zhang Z., Xiao Z. (2012). Image fusion by pulse couple neural network with shearlet. Opt. Eng..

[39] Feng K., Zhang X., Li X. (2011). A novel method of medical image fusion based on bidimensional empirical mode decomposition. J. Converg. Inf. Technol..

[40] Zhan K., Zhang H., Ma Y. (2009). New spiking cortical model for invariant texture retrieval and image processing. IEEE Trans. Neural Netw..

[41] Wang R., Wu Y., Ding M., Zhang X. Medical image fusion based on spiking cortical model. Proceedings of the SPIE 8676, Medical Imaging 2013: Digital Pathology.

[42] Chen J., Shan S., He C., Zhao G., Pietikäinen M., Chen X., Gao W. (2010). WLD: A robust local image descriptor. IEEE Trans. Pattern Anal. Mach. Intell..

[43] The Whole Brain Atlas. http://www.med.harvard.edu/aanlib/home.html.

[44] Image Fusion Toolbox. http://www.metapix.de/toolbox.html.

[45] NSCT-SF-PCNN-ImageFusion-Toolbox. http://dspace.xmu.edu.cn/dspace/handle/2288/8332.

[46] MST_SR_Fusion_Toolbox. http://home.ustc.edu.cn/~liuyu1/publications/MST_SR_fusion_toolbox.

[47] Qu G., Zhang D., Yan P. (2002). Information measurement for performance of image fusion. Electron. Lett..

[48] Xydeas C.S., Petrovic V. (2000). Objective image fusion performance measure. Electron. Lett..

[49] Wang Z., Bovik A.C., Sheikh H.R., Simoncelli E.P. (2004). Image quality assessment: From error visibility to structural similarity. IEEE Trans. Image Process..

[50] Yang C., Zhang J., Wang X., Liu X. (2008). A novel similarity based quality metric for image fusion. Infusion Fusion.

[51] Piella G., Heijmans H. A new quality metric for image fusion. Proceedings of the IEEE International Conference on Image Processing (ICIP).

[52] Cvejic N., Loza A., Bull D., Canagarajah N. (2005). A similarity metric for assessment of image fusion algorithms. Int. J. Signal Process..

[53] Wang Z., Bovik A.C. (2002). A universal image quality index. IEEE Signal Process. Lett..

